# RCAN1.4 attenuates renal fibrosis through inhibiting calcineurin-mediated nuclear translocation of NFAT2

**DOI:** 10.1038/s41420-021-00713-8

**Published:** 2021-10-27

**Authors:** Jianjian Zhang, Hui Chen, Xiaodong Weng, Hao Liu, Zhiyuan Chen, Qin Huang, Lei Wang, Xiuheng Liu

**Affiliations:** 1 Department of Urology, Renmin Hospital of Wuhan University, Wuhan, 430060 Hubei China; 2 Department of Obstetrics and Gynecology, Renmin Hospital of Wuhan University, 430060 Wuhan, Hubei China

**Keywords:** Cell signalling, Apoptosis

## Abstract

Chronic kidney disease (CKD) is thus deemed to a global health problem. Renal fibrosis, characterized by accumulation of extracellular matrix (ECM) components in the kidney, is considered a common pathway leading to CKD. Regulator of calcineurin1 (RCAN1), identified as a competitive endogenous inhibitor of the phosphatase calcineurin, participates in ECM deposition in various organs. However, the role of RCAN1 in renal fibrosis remains unclear. Here, unilateral ureteral obstruction (UUO), a well-known model to induce renal fibrosis in vivo, was performed on mice for a week. To overexpress RCAN1.4 in vivo, recombinant adeno-associated virus 9-packed RCAN1.4 over-expression plasm was employed in mice kidney. Lentivirus-packed RCAN1.4 over-expression plasm was employed to transfer into HK-2 and NRK-49F cells in vitro. The results indicated that RCAN1.4 expression was impaired both in UUO-induced renal fibrosis in vivo and TGF-β1-induced renal fibrosis in vitro. However, knocking in of RCAN1.4 suppressed the production of extracellular matrix (ECM) both in vivo and in vitro. Furthermore, in vitro, the apoptosis-related proteins, including the ratio of Bax/Bcl-2 and cleaved-caspase3, were elevated in cells transfected with RCAN1.4 overexpression plasmid. In addition, we found that RCAN1.4 could rugulated NFAT2 nuclear distribution by inhibiting calcineurin pathway. So overexpression of RCAN1.4 could reverse renal fibrosis, attenuate ECM related protein accumulation, promote apoptosis of myofibroblast via inhibiting Calcineurin/NFAT2 signaling pathway. Taken together, our study demonstrated that targeting RCAN1.4 may be therapeutic efficacy in renal fibrosis.

## Introduction

Chronic kidney disease (CKD) is the 16th cause of death worldwide [1]. The incidence rate of CKD is estimated at 9.1% and thus deemed to a global health problem [2]. Once it irreversibly leads to end-stage renal disease (ESRD), only dialysis and kidney transplantation are available [3, 4]. Regardless of the initial causes, renal fibrosis is considered a common pathway leading to ESRD [3, 5]. The main histopathological features of renal fibrosis are characterized by activation of interstitial fibroblasts, excessive accumulation of extracellular matrix (ECM) in the renal interstitium, leading to a progressive loss of normal renal structure [6, 7]. It has been confirmed that transforming growth factor β1 (TGF-β1), which is secreted from renal tubule cells, is identified as the most important cytokine in the progress of renal fibrosis [8, 9]. Importantly, activated renal interstitial fibroblasts, which are called myofibroblasts and are characterized by the over-expression of α-smooth muscle actin (α-SMA) and type I collagen (COL1a1), can be induced by TGF-β1 [6, 10, 11]. Therefore, targeting for myofibroblasts by inhibiting their activation and inducing their apoptosis might be an attractive strategy to protect against renal fibrosis.

Regulator of calcineurin1 (RCAN1, also referred to as Dscr1/Mcip1) is located on chromosome 21 and composed of four different transcripts [12, 13]. In human tissue, three main RCAN1 isoforms (RCAN1.1, RCAN1.2, and RCAN1.4) are detected depending on promoter usage [14]. Nevertheless, RCAN1.4 has been identified as a competitive endogenous negative regulator of the phosphatase calcineurin which is associated with the activation of nuclear factor of activated T cells (NFAT) [15, 16]. Furthermore, several stimulis of inflammatory cytokines, including TGF-β1, vascular endothelial growth factor (VEGF), reactive oxygen species (ROS), could induce the transcription of RCAN1.4 [17–19]. However, the role of RCAN1.4 in renal fibrosis remains unclear. So far, our studies try to investigate the critical role of RCAN1.4 in renal interstitial fibroblasts activation and kidney fibrosis.

Calcineurin, a calcium-dependent phosphatase, can modulate podocyte and glomerular function in kidney [16]. It has been reported that it is involved in TGF-β1-mediated ECM accumulation in diabetic hearts and liver fibrosis [20, 21]. When Calcineurin inhibitors applied, glomerular disease could be improved [22]. In addition, Calcineurin plays a vital role in regulating apoptosis, tumor process, and metastasis [23, 24]. Calcineurin has many substrates consisting of the transcription factor NFAT, which is dephosphorylated by activated Calcineurin [19]. Furthermore, the expression of downstream genes, including Insulin-like Growth Factor-1 (IGF-1), are regulated by calcineurin/NFAT [25]. Hence, we supposed that, as an endogenous negative regulator of Calcineurin, RCAN1.4 had a critical potential role in regulating ECM accumulation through Calcineurin /NFAT signaling pathway.

In this study, we investigated the mechanism of RCAN1.4 in regulating renal interstitial fibroblast activation and renal fibrosis development through Calcineurin/NFAT in unilateral ureteral obstruction (UUO) mice and in vitro. Also, we demonstrated whether RCAN1.4 could be a potential therapeutic target for patients with CKD.

## Results

### RCAN1.4 expression was down-regulated in vivo and vitro

**Fig. 1:**
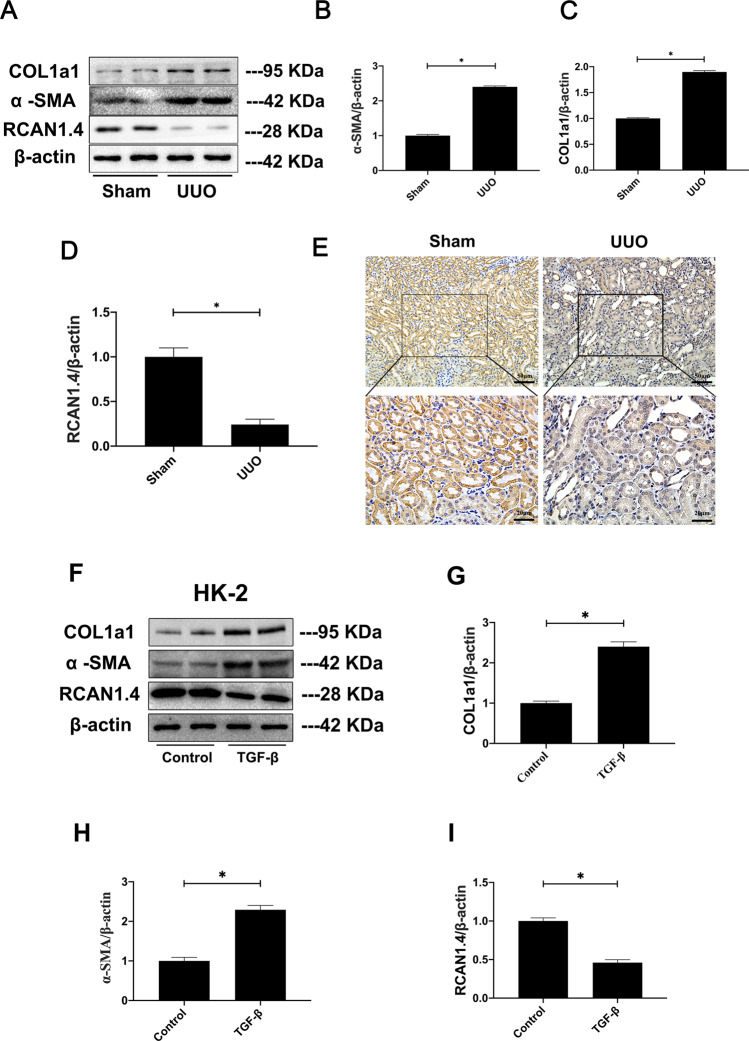
RCAN1.4 expression was down-regulated in vivo and vitro. Western blotting analysis was performed after collecting kidney lysates. **A**, **F** Representative blots of RCAN1.4, COL1a1, α-SMA, and β-actin; **B**, **H** α-SMA/β-actin ratio; **C**, **G** COL1a1/β-actin ratio; **D**, **I** RCAN1.4/β-actin ratio; **E** Immunohistochemistry signals of RCAN1(Scale bars, 20 μm and 50 μm). The results are expressed as the mean ± standard error of the mean (S.E.M.) for 3–4 independent experiments. *p < 0.05, as indicated.

To determine the role of RCAN1.4 in vivo and vitro, we firstly examined the expression levels of RCAN1.4 in unilateral ureteral obstruction (UUO) mice, which was a well-accepted mouse model of renal fibrosis. As shown in Fig. 1A–D, the protein levels of RCAN1.4 were significantly decreased while COL1a1 and α-SMA were elevated in UUO group (*P* < 0.05), compared with the sham group. As displayed in Fig. 1E, immunohistochemical staining showed that the signal of RCAN1 was decreased in the fibrotic kidney tissue of UUO mice compared with the sham group.

RCAN1.4 expression was also decreased in HK‐2 cells that were treated with TGF-β1 (5 ng/mL) in vitro for 24 h. As revealed in Fig. 1F, I, the expression of RCAN1.4 protein was significantly reduced by TGF-β1 stimulation. As shown in Fig. 1F–H, enhanced expression of COL1a1 and α-SMA were detected in TGF-β1-treated HK-2 cells. The result of experiment in vitro was in correspondence with that of experiment in vivo, illustrating that RCAN1.4 was down-regulated in vitro as well and might be one of the major causes implicated in kidney fibrogenesis.

### Recombinant adeno-associated virus-mediated overexpression of RCAN1.4 attenuated renal fibrosis in UUO mice

**Fig. 2:**
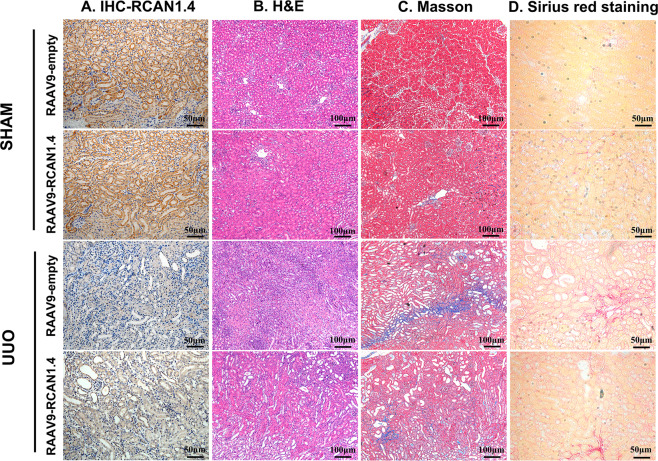
Forced RCAN1.4 overexpression by rAAV9-packed RCAN1.4 over-expression plasm alleviated UUO-induced renal fibrosis. **A**–**D** Representative RCAN1 (Scale bars, 50 μm), H&E (Scale bars, 100 μm), Masson (Scale bars, 100 μm), Sirius red (Scale bars, 50 μm) of kidney interstitium tissue.

**Fig. 3:**
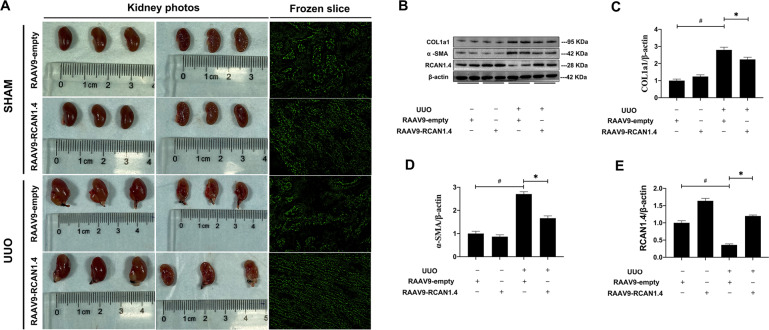
Forced RCAN1.4 overexpression by rAAV9-packed RCAN1.4 over-expression plasm alleviated UUO-induced renal fibrosis. **A** Macroscopic observation of fresh kidney tissues by camera and fluorescence microscope; **B** Representative blot of RCAN1.4, COL1a1, α-SMA, and β-actin; **C** COL1a1/β-actin ratio; **D** α-SMA/β-actin ratio; **E** RCAN1.4/β-actin ratio. The results are expressed as the mean ± standard error of the mean (S.E.M.) for 3–4 independent experiments. *p < 0.05, ^#^*p* < 0.05, as indicated.

To assess whether the decreased expression of RCAN1.4 was associated with the development of renal fibrosis, rAAV9-RCAN1.4 was used to infect the kidney of mice. Immunohistochemical staining showed that RCAN1.4 expression was increased in the rAAV9-RCAN1.4-transfected kidney compared to rAAV9-empty vector-treated renal tissue (Fig. 2A). H&E, Masson staining, and Sirius red staining were performed to validate the pathological changes. As shown in Fig. 2B, comparing with Sham groups, glomerular fibrosis with cystic degeneration, renal tubular inflammatory cell infiltration, and interstitial renal tubular widening were observed in kidney insulted by UUO treatment. Most importantly, overexpression of RCAN1.4 in kidney tissue reduced infiltration of inflammatory cells in UUO group. As revealed in Fig. 2C, D, compared with Sham group, collagen fibrils were extensively displayed in UUO group indicating increased ECM deposition in interstitial space of kidney. Meanwhile, RCAN1.4 overexpression reduced the area of fibrotic tissue induced by UUO. The eGFP signal in rAAV9-RCAN1.4 transfected kidney (Fig. 3A) indicated a successful infection of the virus. As shown in Fig. 3A, the kidneys in UUO groups are visibly larger in size and more swollen. Furthermore, after incision along the coronal plane of the kidney, the renal cortex became thinner and the renal pelvis and calyces became extremely dilated in UUO groups. In accordance with the pathological changes, the protein levels of COL1a1 and α-SMA were significantly increased in UUO group (Fig. 3B–E). However, UUO-mediated upregulated expression levels of hallmarks of EMT were significantly reduced in RCAN1.4 overexpression fibrotic mouse model group. These results further confirmed that overexpression of RCAN1.4 in vivo could attenuate kidney injury and ECM accumulation. Collectively, these data indicated that the upregulation of RCAN1.4 partially protected against UUO-induced kidney fibrosis in mouse and appeared to be a promising therapeutic target.

### Over-expression of RCAN1.4 alleviated kidney fibrosis and promoted apoptosis of TGF-β1-stimulated NRK-49F in vitro

**Fig. 4:**
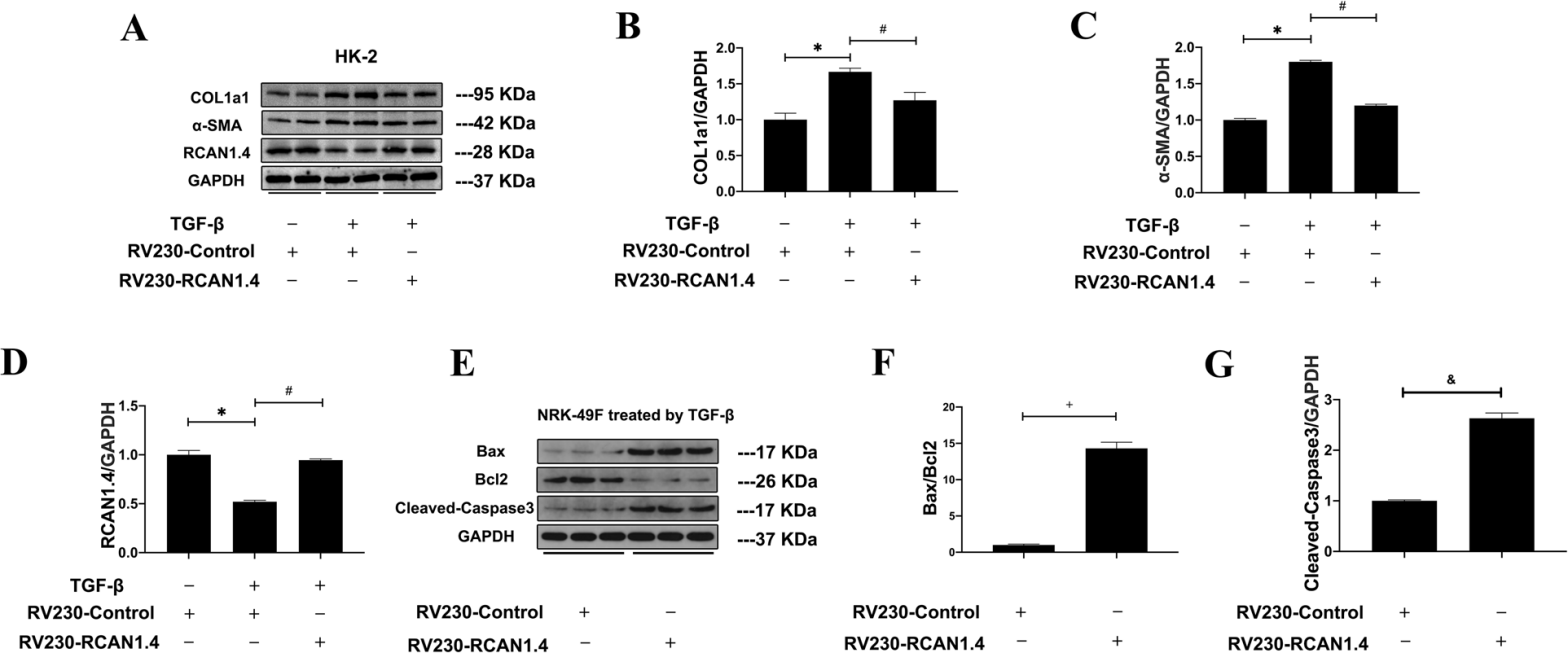
Affection of overexpression of RCAN1.4 in HK-2 cells and NRK-49F cells. **A** Representative blots of RCAN1.4, COL1a1, α-SMA, and GAPDH; **B** COL1a1/GAPDH ratio; **C** α-SMA/GAPDH ratio; **D** RCAN1.4/GAPDH ratio; **E** Representative blots of Bax, Bcl-2, cleaved-caspase3, and GAPDH; **F** Bax/Bcl-2 ratio; **G** cleaved-caspase3/GAPDH; *p < 0.05, ^#^*p* < 0.05, ^+^*p* < 0.01, ^&^*p* < 0.01, as indicated. The results are expressed as the mean ± standard error of the mean (S.E.M.). for 3–4 independent experiments.

The active expression of RCAN1.4 was inhibited after suffering UUO-induced renal fibrosis in vivo and TGF-β1-induced renal fibrosisin vitro. To evaluate whether the high expression of RCAN1.4 was associated with the protection of renal fibrosis damage and the underlying mechanisms, we used RV230-RCAN1.4 plasmid to overexpress RCAN1.4 in HK-2. As shown in Fig. 4A–D, the protein levels of RCAN1.4 were significantly decreased while COL1a1 and α-SMA were remarkably elevated in group treated with TGF-β1. After transfected with RV230-RCAN1.4 plasmid, the expression of RCAN1.4 in HK-2 was upregulated, subsequently suppressing the COL1a1 and α-SMA protein. In view of the need for a better understand of the effect of RCAN1.4 on activated renal interstitial fibroblasts during the development of renal fibrosis, NRK-49F (a rat kidney interstitial fibroblast cell line) cells were employed. All groups were pretreated with TGF-β1 (5 ng/mL) for 24 h. As shown in Fig. 4E–G, the apoptosis-related proteins, including the ratio of Bax/Bcl-2 and cleaved-caspase3, were elevated with stable overexpression of RCAN1.4 (compared to group transfected with RV230-control plasmid). These results indicated that ectopic expression of RCAN1.4 significantly promoted apoptosis in myofibroblasts and ameliorated renal fibrosis.

### Silence of RCAN1.4 aggravated kidney fibrosis and further suppressed the apoptosis in vitro

**Fig. 5:**
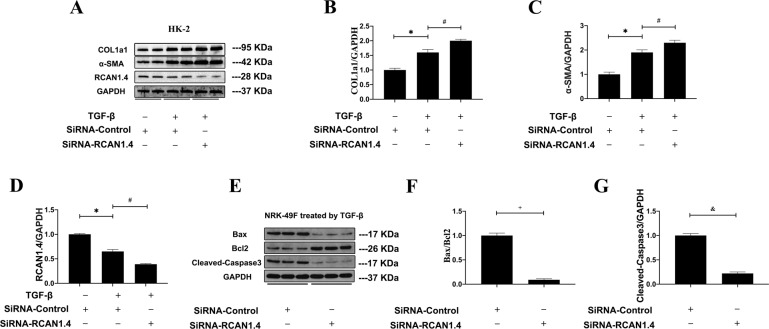
Further influence of RCAN1.4 silencing in HK-2 cells and NRK-49F cells. **A** Representative blots of RCAN1.4, COL1a1, α-SMA, and GAPDH; **B** COL1a1/GAPDH ratio; **C** α-SMA/GAPDH ratio; **D** RCAN1.4/GAPDH ratio. **E** Representative blots of Bax, Bcl-2, cleaved-caspase3 and GAPDH; **F** Bax/Bcl-2 ratio; **G** cleaved-caspase3/GAPDH; *p < 0.05, ^#^*p* < 0.05, ^+^*p* < 0.01, ^&^*p* < 0.01, as indicated. The results are expressed as the mean ± standard error of the mean (S.E.M.). for 3–4 independent experiments.

To further confirm the role of RCAN1.4 in TGF-β1-induced renal fibrosis in vitro, RCAN1.4-RNAi transfection was employed to knock down RCAN1.4 expression in HK-2 cells and NRK-49F cells. As shown in Fig. 5A–D, the protein levels of COL1a1 and α-SMA were increased while RCAN1.4 was significantly deceased in HK-2 cells after stimulated by TGF-β1 (5 ng/mL). Moreover, HK-2 cells, treatment with RCAN1.4-RNAi, showed significantly more upregulation of COL1a1, α-SMA protein expression, and downregulation of RCAN1.4 protein expression. Next, we further explored whether the silence of RCAN1.4 affect the apoptosis in NRK-49F. According to previous study already performed, all cells were treated with TGF-β1 (5 ng/mL) for 24 h. As shown in Fig. 5E–G, the expression of apoptosis-related proteins, including the ratio of Bax/Bcl-2 and cleaved-caspase3, were decreased with further impaired of RCAN1.4 by RCAN1.4-RNAi transfection (*P* < 0.05, compared to group transfected with SiRNA-Control). These data proved that RCAN1.4 silencing attenuated apoptosis in myofibroblasts and exacerbated renal fibrosis.

### RCAN1.4 inhibits calcineurin-NFAT2 signaling in TGF-β1–induced renal fibroblast activation in vitro

**Fig. 6:**
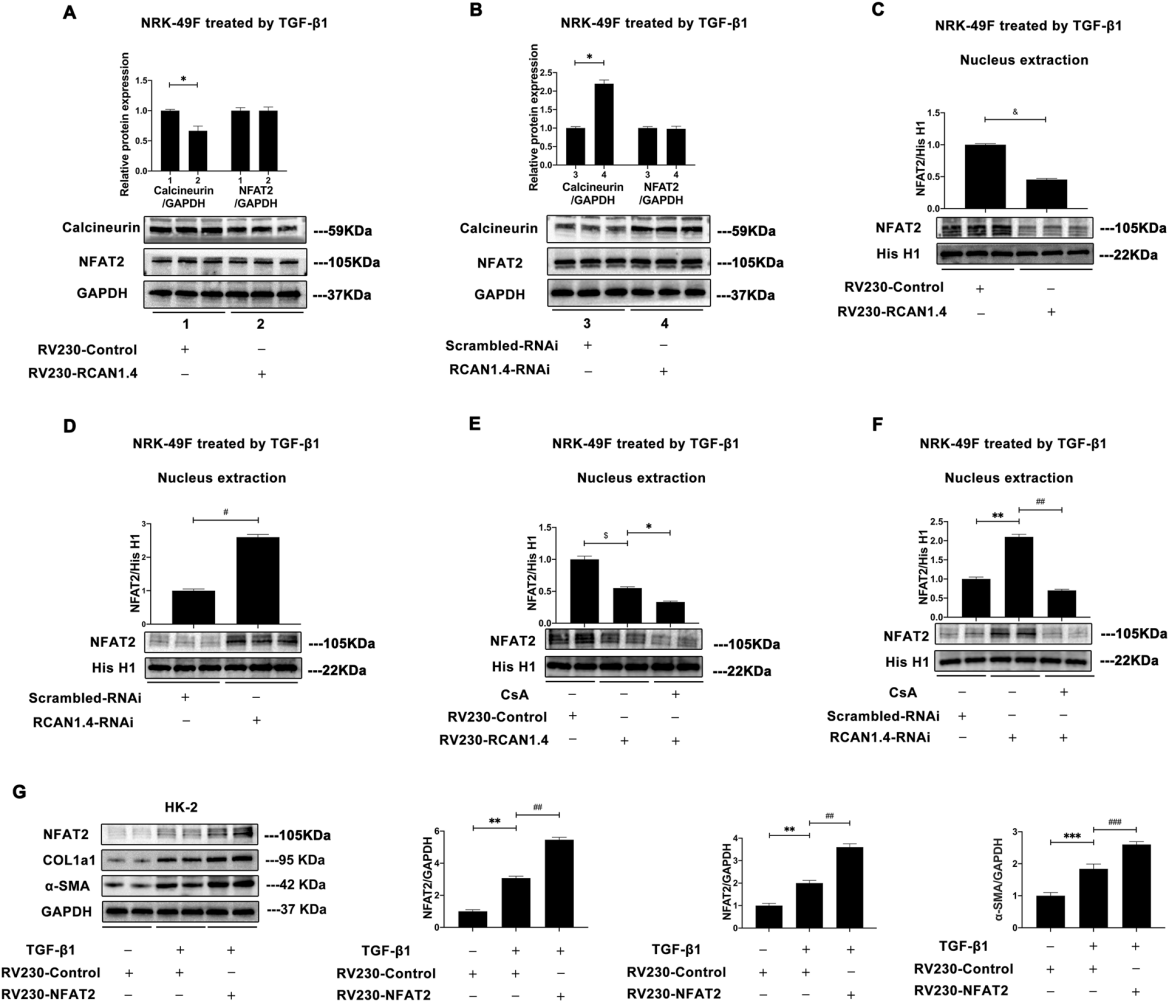
RCAN1.4 negatively regulated the nuclear translocation of NFAT2 in renal fibrosis. **A,** **B** The protein levels of Calcineurin, NFAT2 and GAPDH; **C**–**F** The protein levels of NFAT2 and Histone H1; **G** The protein levels of COL1a1/GAPDH ratio; α-SMA/GAPDH ratio; RCAN1.4/GAPDH ratio; *p < 0.05, **p < 0.05, ***p < 0.05, ^#^*p* < 0.05, ^##^*p* < 0.05, ^###^*p* < 0.05, ^$^*p* < 0.05, ^&^*p* < 0.05, as indicated. The results are expressed as the mean ± standard error of the mean (S.E.M.) for 3–4 independent experiments.

RCAN1 was demonstrated to particularly block nuclear localization and transcriptional activation of NFAT by calcineurin [26, 27]. Consequently, the protein expression of calcineurin was evaluated in NRK-49F cells after transfected with RV230-RCAN1.4 plasmid or RCAN1.4-RNAi. All NRK-49F cells were pretreated with TGF-β1 (5 ng/mL) for 24 h as before. As shown in Fig. 6A, B, Calcineurin protein expression was remarkably reduced after stable knock-in of RCAN1.4, while increased when RCAN1.4 was knocked down in NRK-49F cells. Next, the nuclear accumulation state of NFAT2 was measured by Western blot. Nuclear and cytoplasmic proteins were extracted by Nuclear and Cytoplasmic Protein Extraction Kit. As shown in Fig. 6C, D, overexpression of RCAN1.4 suppressed nuclear translocation of NFAT2, whereas knockdown of RCAN1.4 elevated NFAT2 nuclear distribution in NRK-49F cells. However, total cellular protein expression of NFAT2 was not influenced by RCAN1.4 (Fig. 6A, B). Taken together, these results suggested that the change of NFAT2 nuclear level was attributed to the NFAT2 translocation from cytoplasm to nucleus but not the variation of total NFAT2 expression.

Then, for further investigating the mechanism of RCAN1.4 in regulating NFAT2, NRK-49F cells were treated with cyclosporin A (CsA), a specific calcineurin inhibitor, and NFAT2 nuclear accumulation level was examined. As shown in Fig. 6E, F, the NFAT2 nuclear translocation, upregulated by silenced RCAN1.4, was reversed by CsA treatment. On the contrary, CsA treatment further reduced NFAT2 nuclear level which was downregulated by overexpression of RCAN1.4. Thus, RCAN1.4 regulated NFAT2 nuclear distribution by inhibiting calcineurin pathway. Previous study demonstrated that Calcineurin/NFAT2 signaling pathway was involved in cardiac hypertrophy [27]. To confirm the function of NFAT2 on renal fibrosis, NFAT2 overexpression plasmid was employed in HK-2 cells. As shown in Fig. 6G, forced expression of NFAT2 by RV230-NFAT2 further exacerbated renal fibrosis, reflected by increased protein expression of COL1a1 and α-SMA. All these studies confirmed that RCAN1.4 negatively regulated nuclear translocation of NFAT through inhibiting calcineurin pathway.

### IGF1, regulated by NFAT2, was essential for accumulation of ECM in renal fibrosis

**Fig. 7:**
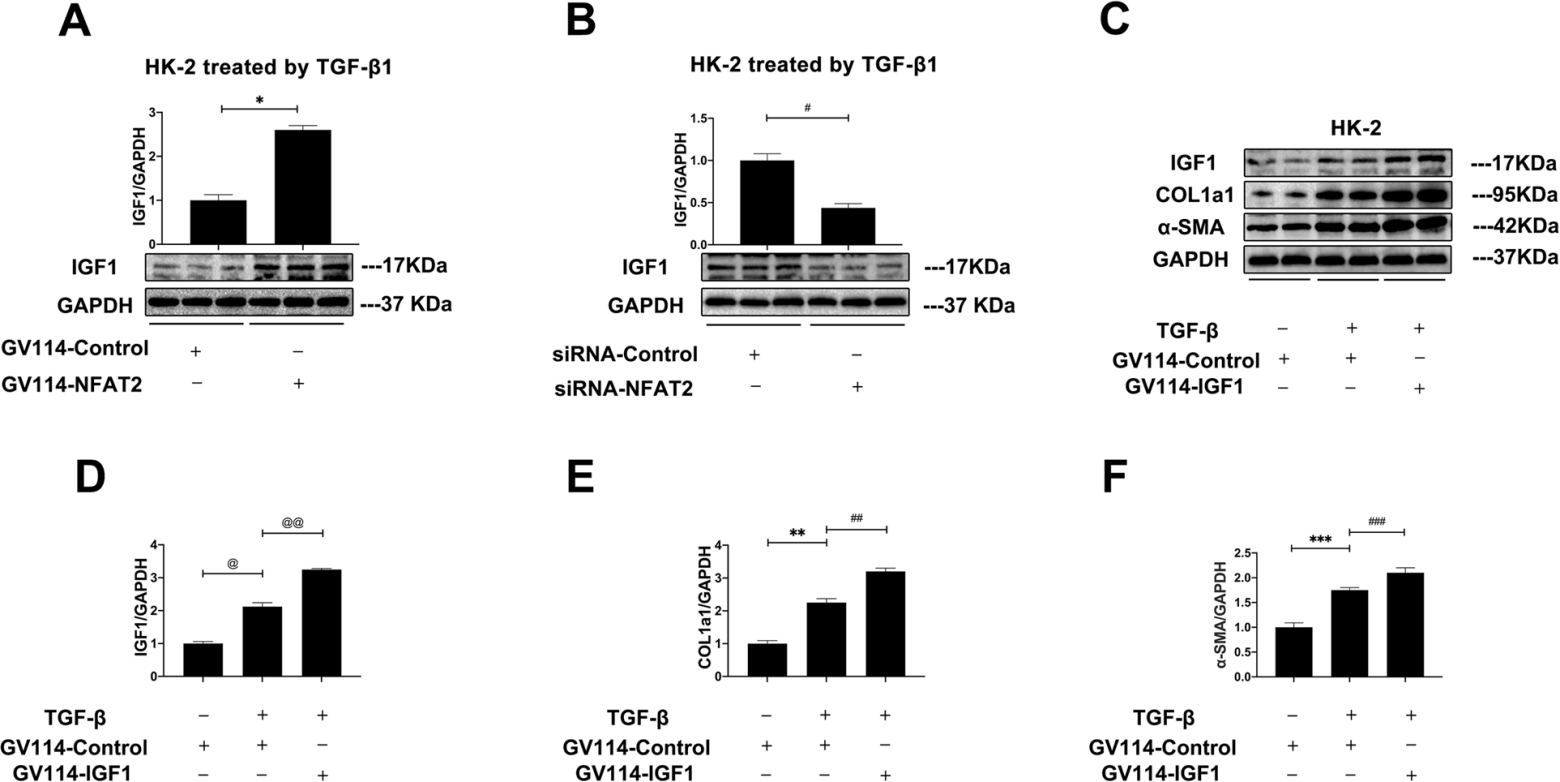
IGF1 was regulated by NFAT2 and was essential for accumulation of ECM in renal fibrosis. **A**, **B** The protein levels of IGF1 and GAPDH; **C** Representative blots of IGF1, COL1a1, α-SMA, and GAPDH; **D** IGF1/GAPDH ratio; **E** COL1a1/GAPDH ratio; **F** α-SMA /GAPDH ratio; *p < 0.05, **p < 0.05, ***p < 0.05, ^#^*p* < 0.05, ^##^*p* < 0.05, ^###^*p* < 0.05, ^@^*p* < 0.05, ^@@^*p* < 0.05, as indicated. The results are expressed as the mean ± standard error of the mean (S.E.M.) for 3–4 independent experiments.

It has been reported that IGF1 expression was directly transactivated by NFAT1 and contributed to the progression of hepatocellular carcinoma [23]. To explore the role of NFAT2 in regulating expression of IGF1, NFAT2 over-expression plasm or siRNA-NFAT2 was employed. All NRK-49F cells were stimulated by TGF-β1 for 24 h. As shown in Fig. 7A, B, overexpression of NFAT2 decreased IGF1 protein levels while silenced NFAT1 expression increase IGF1 protein levels in NRK-49F cells. Then, we treated HK-2 cells with TGF-β1 or GV114-IGF1 to examine the influence of IGF1 expression on renal fibrosis. As shown in Fig. 7C–F, the protein level of IGF1 was upregulated by TGF-β1 stimulation. Moreover, forced expression of IGF1 further aggravated the accumulation of ECM reflected by increased protein expression of COL1a1 and α-SMA. It was preliminarily concluded that IGF1 played a vital role in renal fibrosis.

### RCAN1.4 expression correlated with renal interstitial fibrosis in human kidney biopsy specimens

**Fig. 8:**
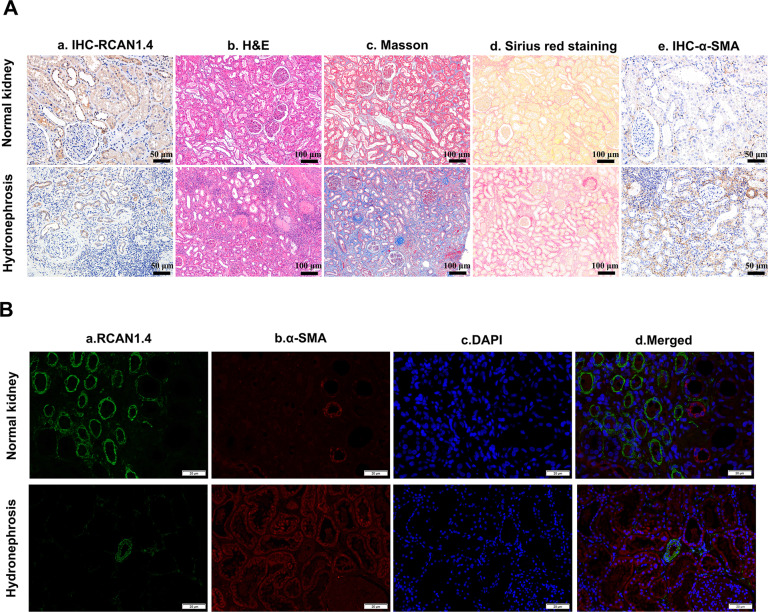
RCAN1.4 expression correlated with renal interstitial fibrosis in human kidney biopsy specimens. **A a**, **e** Immunohistochemistry signals of RCAN1 and α-SMA (Scale bars, 50 μm); **A** **b**, **c**, **d** H&E, Masson, Sirius red of kidney interstitium tissue (Scale bars, 100 μm). **B** RCAN1 and α-SMA protein expression in renal tissue was examinated by immunofluorescence (Scale bars, 20 μm). The results are expressed as the mean ± standard error of the mean (S.E.M.).

Last, to test the RCAN1.4 expression in human renal interstitial fibrosis, we used human kidney biopsy specimens that were obtained from patients with severe hydronephrosis. As shown in Fig. 8A a, immunohistochemistry showed that RCAN1.4 expression was suppressed in severe hydronephrosis. H&E, Masson staining and Sirius red staining were performed to validate the pathological and fibrotic changes. As shown in Fig. 8A b–d, kidney with severe hydrops displayed increased ECM deposition. Moreover, as shown in Fig. 8A e, α-SMA signal indicating induced ECM deposition. Furthermore, the colocalization of RCAN1.4 and α-SMA in both human normal kidney and renal fibrosis tissues were tested by immunofluorescence. As shown in Fig. 8B a–d, RCAN1.4 expression was detected mainly in α-SMA-negetive tubular epithelial cells in normal kidney. In addition, RCAN1.4 expression was almost undetectable in α-SMA-positive area in renal fibrosis tissues. These indicated that RCAN1.4 expression was impaired in human renal interstitial fibrosis.

## Discussion

In this study, we demonstrated that RCAN1.4 expression was down-regulated in renal fibrosis in mouse and in activated renal interstitial fibroblasts. Furthermore, the results showed that TGF-β1 induced renal fibrosis in vivo and in vitro through inhibiting the RCAN1.4 expression. We also observed the novel insights that exogenous forced RCAN1.4 expression could alleviate the injury of renal fibrosis through Calcineurin/NFAT2 signaling pathway.

RCAN1, the specific endogenous inhibitor of Calcineurin, has been reported participating in various physiological functions, including regulating VEGF-mediated tubular morphogenesis in endothelial cells, leading to directed cell migration [28], influencing metabolism and thermogenesis [19], defending against oxidative stress mediated by calcium [29], inhibiting growth, angiogenesis, and metastases of hepatocellular carcinoma [23]. In previous studies, RCAN1 has been demonstrated being involved in neurological disease, cardiovascular problems, and liver dysfunction [30–32]. Nevertheless, there is still poor understanding of RCAN1 in kidney pathologies including renal fibrosis. Jang C et al. reported that RCAN1.4 expression was down-regulated in murine diabetic nephropathy [33]. Li H et al. demonstrated that RCAN1 expression was reduced in HIV-induced glomerular diseases through calcineurin-NFAT pathway [34]. Sang X et al. proved that overexpression of RCAN1.4 can reverse tubulointerstitial fibrosis by regulating the mitochondrial function [35]. In our study, we established the UUO model [36], a universal model for inducing fibrosis on mouse kidney, to confirm the role of RCAN1.4 in renal fibrosis. We could definitely demonstrate that RCAN1.4 expression was impaired after suffering UUO-induced renal fibrosis in vivo and TGF-β1-induced renal fibrosisin vitro. In addition, knocking in of RCAN1.4 accelerated apoptosis of myofibroblast and attenuated renal fibrosis in vitro. As a consequence, RCAN1.4 exhibited a promising therapeutic target on renal fibrosis. In liver fibrosis, RCAN1.4 was decreased by elevated methyltransferases DNMT1 and DNMT3b [21]. Li H et al. reported that epigenetic inhibition of RCAN1 exacerbates podocyte injury in HIV-infected nephropathy [34]. However, it remains to be assessed the deep mechanism of downregulation of RCAN1.4 caused by renal fibrosis.

One of the key roles of RCAN1 was associated with the regulation of Calcineurin /NFAT pathway [37]. NFAT has four subtypes (NFAT1-4) which participate in different physiological activities [38]. Huang B et al. illustrated that RCAN1.4 improved a hypoxia-induced degenerative disc disease through NFATc1 [39]. In hepatocellular carcinoma tissues, RCAN1.4 was reported to inhibit the activation of and subsequently prevented nuclear localization of NFAT1 and restrained the liver cancer cell migration, proliferation [23]. Moreover, Ryeom et al. inferred that there was a delicate balance between RCAN1 and Calcineurin/NFAT in angiogenic cancer [40]. However, which subtype of NFAT produced a marked effect in renal fibrosis and how it was regulated by RCAN1.4 were not clear yet. In our study, RCAN1.4 inhibited the phosphatase activity of calcineurin. Furthermore, overexpression of RCAN1.4, induced by rAAV9-packed RCAN1.4 over-expression plasm, could inactivate Calcineurin which subsequently blocked the nuclear translocation of NFAT2 and could also promote apoptosis of myofibroblast in vitro reflected by increase of related protein levels. To further validate the regulatory role of RCAN1.4 on Calcineurin /NFAT2 signaling pathway, CsA was employed in vitro, reflecting that CsA restored RCAN1.4-mediated inhibition of NFAT2 nuclear translocation.

Previous literatures reported that the downstream signals of NFAT were quite a lot, including Interleukin-2 (IL-2), Interleukin-8 (IL-8), vascular endothelial growth factor (VEGF), chemokine (MMP) etc. [26, 41]. In our study, we found the expression of IGF1 was directly regulated by NFAT2 in myofibroblast induced by TGF-β1. IGF1 was noted to play a crucial role in metastasis and tumor growth of liver cancer [25]. Verrotti A et al. reported that down-regulation of IGF-1 was involved in patients with diabetic nephropathy [42]. Our in vitro study confirmed that forced IGF1 expression could attenuate activation of myofibroblast with elevated ECM-related protein levels. Renal fibrosis, with superfluous deposition of ECM as its hallmark, was the same as fibrosis in other organs [43]. It was a potential strategy to treat fibrosis for slowing down the aging process and decreasing human mortality [44]. However, further studies are required to address on how RCAN1.4 regulates the proliferation and migration of myofibroblast and the underlying mechanism of its function in alleviating the ECM deposition in renal interstitium.

In summary, we provided evidence that RCAN1.4 played a novel role in the progress of renal fibrosis. The expression of RCAN1.4 was attenuated both in UUO-induced fibrotic kidney of mouse and in TGF-β1-induced fibrotic model in vitro. Moreover, overexpression of RCAN1.4 could reverse renal fibrosis, attenuate ECM related protein accumulation, and promote apoptosis of myofibroblast via inhibiting Calcineurin /NFAT2 signaling pathway. Taken together, our study demonstrated that targeting RCAN1.4 may have therapeutic benefits on renal fibrosis.

## Materials and methods

### Animal preparation

Male adult C57BL/6 J mice (20–25 g) were provided by the center of experimental animals in the medical College of our university. The animals were placed in a room with suitable temperature and humidity and got free access to rat edibles and tap water. This experiment was authorized by the Ethics Committee of Renmin Hospital of Wuhan University (No.00012986), and the procedures were carried out adhering to the principles of Animal Care of Wuhan University (Wuhan, China). Mice were acclimated for a week, and then randomly divided into Sham and Unilateral Ureteral Obstruction (UUO) groups (*n* = 7). In UUO group, based on a well-known model to induce renal fibrosis [45], the kidneys of mice were exposed through a midline abdominal incision, and then the left ureter was double-ligated. Mice in Sham group were performed with sham operations without being ligated on left ureter. After one week of establishing the UUO model, renal tissues of mice were harvested [46].

### Recombinant adeno-associated virus-mediated RCAN1.4 over-expression in mice

Recombinant adeno-associated virus (rAAV) is a single-stranded DNA virus which is used for gene delivery [47]. To demonstrate the critical role of RCAN1.4 in the development of renal fibrosis, rAAV9-packed RCAN1.4 over-expression plasm labeled with the green fluorescent protein (GFP), obtained from ViGene Biosciences, Inc (Jinan, China), was employed to over-express RCAN1.4 in mice kidney. After one week of adapting to the environment, mice were slowly injected with rAAV9-packed RCAN1.4 (100ul, 1 × 10^12^ v.g/mL/mouse) through tail mainline. Subsequently, UUO or Sham operation was performed on mice as described above.

### Cell culture and treatment

To discover the role of RCAN1.4 on different cell lines, the human renal tubular epithelial cells (HK-2) and normal rat kidney (NRK)−49F cells, obtained from the American Type Culture Collection (ATCC, Manassas, VA), were employed. They were cultured in Dulbecco’s modified Eagle’s medium (DMEM, Invitrogen, USA) supplemented with 10% Fetal Bovine Serum (FBS, Gibco, USA) at constant-temperature incubator (37 °C, 5% CO_2_, 21% O_2_). To establish the cell model of TGF-β1–induced activation of renal fibroblasts in vitro, 5 ng/mL TGF-β1 was administrated to stimulate the cells for 24 h. Afterwards, all the analyses were carried out on collected cells.

### Transfection of RCAN1.4, IGF1, NFAT3 overexpression plasmid constructs

Lentivirus-packed RCAN1.4 over-expression plasm labeled with the GFP (RV230-RCAN1.4, ViGene Biosciences, Inc, Jinan, China) was employed to transfer into HK-2 and NRK-49F cells at a concentration of 1 × 10^7^TU/ml. After 8 h, the cells were observed and the medium was replaced by complete medium. Forty-eight hours later, cells were screened by puromycin (Sigma-Aldrich, St. Louis, MO, USA). All plasmid constructs were obtained from ViGene Biosciences, Inc (Jinan, China). Cells (3 × 10^5^/mL) were seeded in 6-well plates and transfected with the NFAT2 (RV230-NFAT2) or IGF1 (GV114-IGF1) plasmid and control constructs mixed with Lipo2000 transfection reagent (Invitrogen, Carlsbad, CA, USA). After changing the medium, cells were cultured for an additional 24 h before carrying out further experiments.

### Small interfering RNA (SiRNA) transfection in vitro

For RCAN1.4, NFAT2, HK-2 cells and NRK-49F cells were transfected with related siRNAs obtained from ViGene Biosciences, Inc (Jinan, China). Twenty-four hours later, the culture medium was replaced by DMEM containing 10% FBS and TGF-β1 (5 ng/mL). The following siRNA sequences were used:RCAN1.4-siRNA (sense: 5′- UUGCUCAGACCUUACACAUAGTT-3′ and antisense: 5′- CUAUGUGUAAGGUCUGAGCAAT-3′); RCAN1.4-siRNA (sense: 5′- CGUGUGUGAGAGUGACCAAGA-3′ and antisense: 5′- UUGGUCACUCUCACACACGUG-3′); NFAT2-siRNA (sense: 5′-GCAUGUGUGUACAUAUCUAGG -3′ and antisense: 5′- UAGAUAUGUACACACAUGCAA-3′); siRNA-control with scrambled sequence: (sense: 5′-UUCUCCGAACGUGUCACGUTT-3′ and anti-sense: 5′ACGUGACACGUUCGGAGAATT-3′).

### Histopathology and immunohistochemistry

Renal tissue samples were collected from mice and were fixed in 4% paraformaldehyde, embedded in paraffin and 4 µm thick serial sections were obtained. Then, the sections were deparaffinized, hydrated, and stained with hematoxylin and eosin (H & E) in order to assess histopathological kidney injury and Sirus-red and Masson’s trichrome staining for observing collagen deposition. On the basis of the procedure described in previous research, immunohistochemical staining of RCAN1.4 was performed [48]. The following primary antibody of rabbit anti-RCAN1 (1:200 dilution, pH 8.0, Sigma-Aldrich, USA) was used. The stained tissues were viewed by Ortho microscope (OLYMPUS, Tokyo, Japan).

### Immunofluorescent double-staining

For tissue, frozen sections (4 μm thick) of renal tissue were washed three times/5 min with PBS at room temperature. Slides were blocked using 5% bovine serum albumin for 30 min. For immunofluorescent staining, primary antibodies of RCAN1 antibody (1:200 dilution, Sigma-Aldrich, USA) and the α-SMA antibody (1:200 dilution, Bioss, China) and fluorescent-conjugated secondary antibodies were applied to the sections after blocking with preimmune serum from the same species for the secondary antibody. All stained sections were viewed by fluorescent confocal microscopy (Nikon, Tokyo, Japan).

### Main reagents and antibodies

The primary antibodies to COL1a1 (bs10423R) and α-SMA (bs0189R) were purchased from Bioss (Beijing, China), NFAT2 (ab2796) antibody, Calcineurin (ab52761) antibody, bcl2(Ab59348) antibody, and Bax (Ab32503) antibody were produced by Abcam (Cambridge, UK), RCAN1 (D6694) antibody was obtained from Sigma Aldrich (St. Louis, USA), C-Caspase 3 antibody was obtained from Affinity (Jiangsu, China), IGF1 antibody (28530-1-AP), GAPDH (60004-1-Ig), β-actin (20536-1-AP), and Histone H1 (17510-1-AP) antibodies were purchased from Proteintech (Wuhan, China). Goat anti-mouse and goat anti-rabbit secondary antibodies were purchased from Boster Biological Technology (Wuhan, China). For critical chemicals and commercial assays, recombinant human TGF-β1 (100-21) was obtained from Peprotech (New Jersey, USA), Nuclear and Cytoplasmic Protein Extraction Kit (P0027) was purchased from Beyotime Biotechnology (Shanghai, China). Cyclosporin A (CsA) was obtained from Sigma Aldrich (St. Louis, USA).

### Western blot analysis

Samples of mouse kidney were collected and snap-frozen in liquid nitrogen. We then used these tissues to extract total proteins. The Bicinchoninic Acid (BCA) method was performed to quantify protein levels prior to Western blotting. In brief, protein samples were separated on sodium dodecyl sulfate-polyacrylamide gel electrophoresis (SDS-PAGE) gels and then transferred to a polyvinylidene difluoride (PVDF) membrane. Subsequently, PVDF membranes were blocked with 5% non-fat milk for 2 h and then incubated at 4°C overnight with specific antibodies against RCAN1 (1:500), COL1a1 (1:500), α-SMA (1:500), NFAT2 (1:1000), Calcineurin (1:1000), C-Caspase (1:1000), bcl2 (1:1000), Bax (1:2000), Histone H1 (1:1000), GAPDH (1:1000), and β-actin (1:100). After overnight, the PVDF membranes were washed three times with TBST and then incubated with secondary antibody for 2 h at 37 °C. Specific bands were detected by ECL™ (Beijing Pierce Biotechnology, China) and band densities were quantified using Image J software (NIH, Bethesda, MD, USA).

### Clinical sample collection

Kidney specimens were obtained from patients who were diagnosed for severe hydronephrosis at Renmin Hospital of Wuhan University (Approval No.WDRY2019-K056). This study was approved by the Ethics Committee of Wuhan University.

### Statistical analysis

All data are expressed as mean ± standard error of the mean (SEM). Statistical analyses involved one-way analysis of variance (ANOVA) and the Student–Newman–Keuls test. *P*-value <0.05 was accepted as statistically significant.

## Data Availability

The datasets used and/or analyzed during the current study are available from the corresponding author on reasonable request.
